# The Dietary in Indigestion

**Published:** 1886-04

**Authors:** J. Milner Fothergill

**Affiliations:** M. D. Edin.


*From the Journal of Reconstruction.*

### THE DIETARY IN INDIGESTION.

BY J. MILNER FOTHERGILL, M. D., EDIN.

When I hear medical men denouncing a regulated dietary in indigestion, my surprise is excited. Is it a malady to be combatted by drugs alone? I do not think anyone will support that proposition, Medicinal agents are not without their value; but the medicinal treatment of indigestion is surely but ancillary to the dietetic management. That a regulated dietary is too often a restricted dietary—so restricted indeed that the patient is practically half-starved—may be admitted. But need a regulated dietary necessarily be a restricted one? I opine not; if the matter of the dietary of the dyspeptic be given a little more attention.

And for this it is well to keep the physiology of indigestion in mind. Digestion is solution by hydration so that the carbo-hydrates and albuminoids may pass through the wall of the alimentary canal; after which they are de-hydrated—else they would pass out by the kidney, giving glycosuria and peptonaria and leaving the body unfed. But a preliminary to solution is disintegration. If mastication be not properly performed the "lumps" of food find their way into the stomach and offend it.

Pastry, pieces of hard potato, cheese, are notorious offenders. The solvent action of the gastric juice can exercise no disintegrating effect upon the substances, while they act as irritants and set up pain. A piece of meat comparatively unchewed is less objectionable because the gastric juice acting upon the connective tissue allows the muscular fibrillæ to fall asunder. But even with muscular fibre there is a wide difference. Pork and veal are hard meats, and not readily falling to pieces in the stomach under the action of the gastric juice are held, and rightly too, to be indigestible. On the other hand a thin slice of well boiled ham, cut a cross the fibre is very digestible So is the loose fibre of a sheep's head. This is the mechanical aspect of the digestibility of food. Hard stringy meat is very indigestible. So are ill-cooked vegetables, and especially the cruciferæ, so are hard boiled eggs.

Fish, and especially white fish, whose fibres very readily fall to pieces, are in repute with dyspeptics for obvious reasons. Fish which are fatty, are indigestible (because the fat resists the action of the gastric juice) as the flesh of the salmon, the mackerel and the herring. The short fibre of the whiting, "the chicken of the sea," makes this fish especially digestible. Then come the flat-fishes, the haddock and the cod. They all are best boiled, for if fried, care is requisite that the flesh be not soaked in fat—when it is highly indigestible. There are few more indigestible matters than a fried sole which has not been skillfully cooked. And the same holds good of birds. Chicken and game are digestible, while the duck and goose, greasy-fibred meats are as certainly indigestible.

Potatoes have an evil reputation, but that again is largely a matter of cooking. A potatoe which is imperfectly cooked has a hard centre. A "stone" an Irishman calls it—and if palpable pieces of such hard indigestible matter be swollowed gastric distress is the intelligible result. But if the potato be well cooked and put through a seive it ceases to be indigestible from the "mechaniaal point of view." It is the question of disintegration which militetes against vegetables, and uncooked fruit. Peices of hard apples are notoriously indigestible; while a baked apple will sit lightly on the most irritable stomach. The flesh of the grape is in great repute in all conditions of gastric irritability and debility whether primary or secondary to some general sickness.

Fat is an offense to a susceptible stomach, even as liquid fat floating about in it; but still more as lumps of fat upon which the stomach can exercise no solvent influence. Hence many persons, children and adults, reject sweet pieces of fat, and (after the meal) take some fishy oil. As the digestion of fat does not commence till the food has left the stomach, it is not well to give fat till its "time draws nigh." Thin stale bred with butter rubbed well in and doubled is much more digestible than the same bread cut thick with a stout layer of butter plastered over it.

Pastry, when fat and flour are well rubbed together, form a most indigestible compóund resisting all disintegration except mastication. Suet puddings and dumplings also are indigestible.

On the other hand milk puddings, especially if made without an egg, are in repute, and not without reason for dyspeptics. They are light and sit easily on the stomach, the farinaceous matter being readily disintegrated, and what escapes disintegration is soft, and does not give offence to the stomach.

There is another matter not of accult but of microscopic disintegration, or actual solution which has yet to be discussed—a matter of vital importance. As savage man sat grinding the cereals which form so large a factor in human food, the action of the jaws produced a free flow of saliva, and as fast as the finer particles were broken off the seed, by the crunching of the teeth, diastase of the saliva converted the insoluble starch into the soluble dextrine and grapesugar.

The toil of the miller produces disintegration and relieves the jaws of much of the labor. But disintegration is only the precursor of solution. The starch granule rem.ins. By heat the cook cracks the starch granule so that the solvent diastese can readily act upon it. So far, so good: but heat does something more. It has an actual solvent action and heat will, if sufficient, cause conversion of starch into dextrine. A thoroughly well baked flour if subjected to the iodine test under a microscope will readily show this.

When a large quantity of raw unconverted starch enters the stomach it is a burden to that viscus. The gastric juice has no effect upon starch and the starch granules merely embarass the action of the stomach until they find their way out of it by the pyloric ring —and sometimes by the way they entered, viz., the gullet. Undigested starch hampers the stomach and makes the labor of that viscus a painful toil to it. New bread is a gross mechanical irritant, resisting disintegration. The impediment caused by isolated but numerous starch granules is another matter. Biscuits and crackers if insufficieutly masticated cause indigestion. So do cakes which have not long been exposed to heat. The cakes which ara held in such favor by the breakfast table in American households, have been regarded as indigestible, and a glance at the American cooking book explains why. These cakes are exposed to heat for from thirty to forty minutes only. [The language of England sometimes requires translation. For cakes read rolls, and for biscuit read cracker.—Ed.] A good biscuit or loaf is much longer in the oven. Potatoes are indigestible as ordinarily eaten, because they are not long enough exposed to heat. But if well mashed potatoes be put into the oven to brown, or be placed before the fire for that purpose, the longer exposure to heat tells upon the starch-conversion.

Hominy that is well-boiled or subjected to the final heating process of cooking is decidedly digestible. Cereals that have been steam-cooked are in repute with dyspeptics either for adding to meat teas, or for preparing milk-puddings. Some cooks who have to cater for dyspeptics boil all their rice, sago, and tapioca thoroughly before making these up with milk for a milk-pudding. In Germany pearl-barley thoroughly well boiled and passed through a sieve is in request as an addition to meat teas for invalids, The porridge of Scotland being made with coarse oat meal is boiled a long time, while in England a short boil is enough with the fine ground oatmeal in vogue there.

The advantage of the numerous prepared foods—whether babies' food or invalids' foods—which are all more or less compounds of starch which has been to a certain extent predigested either by ba. king or the malting process, lies in their ready digestibility. A touch of saliva is enough to complete the conversion of such carbo-hydrates and the soluble matters pass our of the alimentary canal, and the stomach is not burdened with a weight of undigested starch impeding its work.

Gross and fine disintegration of food are cardinal matters in the dietary of dyspeptics.

Mastication must be perfect else gross particles embarrass the stomach. Starch granules which have escaped the saliva interfere with the solvent action of the gastric juice on albuminoids. The dietary of dyspeptics must be conducted on the above lines; and if the dyspeptic were properly informed he could find a sufficient variety of food; but if he be told to diet himself upon a number of articles of food he soon begins to loathe them and often goes without food sooner than partake of them.

Of course there are dyspeptics and dyspeptics! Some only require to give a sufficiency of time to the process of mastication to be free from suffering. Others must eschew pastry, veal and pork. Others again have to abandon solid meat and vegetables and adhere to meat broths, with cooked starch, malt extracts, malted preparations, milk puddings and fish. When the stomach has been outraged or offended care is requisite for its restoration. When there is present a condition of general exhaustion food will disagree which ordinarily can be taken with impunity. When a condition of acute indigestion is set up a very careful dietary for a few days is directly curative.

Ready disintegration and solubility of food constitute the base line of the dietetic treatment of indigestion.

---

**Borax as an Internal Disinfectant.**—In the *Union Medicale*, Dr. Cyon confirms the statement made by Dumas in 1878, that borax is possessed of most valuable antiseptic powers. Independently of its value for the preservation of food, it is a great preventive of infectious diseases, and may be employed internally to ward off epi. demics. It may be taken for months or years with impunity, and constitutes a valuable prophylatic. Dr. Cyon states that it is a remarkable fact in all epidemics of cholera the workmen in boracic acid factories have always escaped the disease. The usual dose is five or six grammes (75 to 90 grains) daily, taken for an indefinite time.

